# Hemorrhage-Adjusted Iron Requirements, Hematinics and Hepcidin Define Hereditary Hemorrhagic Telangiectasia as a Model of Hemorrhagic Iron Deficiency

**DOI:** 10.1371/journal.pone.0076516

**Published:** 2013-10-16

**Authors:** Helen Finnamore, James Le Couteur, Mary Hickson, Mark Busbridge, Kevin Whelan, Claire L. Shovlin

**Affiliations:** 1 National Heart and Lung Institute, Cardiovascular Sciences, Imperial College London, London, United Kingdom; 2 Nutrition and Dietetics, Imperial College Healthcare NHS Trust, London, United Kingdom; 3 Clinical Chemistry, Imperial College Healthcare NHS Trust, London, United Kingdom; 4 Diabetes and Nutritional Sciences Division, King’s College London, School of Medicine, London, United Kingdom; 5 National Heart and Lung Institute, Cardiovascular Sciences, Imperial College London, London, United Kingdom; 6 Respiratory Medicine, Imperial College Healthcare NHS Trust, London, United Kingdom; 7 University of Liverpool Medical School, Liverpool, United Kingdom; Royal College of Surgeons, Ireland

## Abstract

**Background:**

Iron deficiency anemia remains a major global health problem. Higher iron demands provide the potential for a targeted preventative approach before anemia develops. The primary study objective was to develop and validate a metric that stratifies recommended dietary iron intake to compensate for patient-specific non-menstrual hemorrhagic losses. The secondary objective was to examine whether iron deficiency can be attributed to under-replacement of epistaxis (nosebleed) hemorrhagic iron losses in hereditary hemorrhagic telangiectasia (HHT).

**Methodology/Principal Findings:**

The hemorrhage adjusted iron requirement (HAIR) sums the recommended dietary allowance, and iron required to replace additional quantified hemorrhagic losses, based on the pre-menopausal increment to compensate for menstrual losses (formula provided). In a study population of 50 HHT patients completing concurrent dietary and nosebleed questionnaires, 43/50 (86%) met their recommended dietary allowance, but only 10/50 (20%) met their HAIR. Higher HAIR was a powerful predictor of lower hemoglobin (p = 0.009), lower mean corpuscular hemoglobin content (p<0.001), lower log-transformed serum iron (p = 0.009), and higher log-transformed red cell distribution width (p<0.001). There was no evidence of generalised abnormalities in iron handling Ferritin and ferritin^2^ explained 60% of the hepcidin variance (p<0.001), and the mean hepcidinferritin ratio was similar to reported controls. Iron supplement use increased the proportion of individuals meeting their HAIR, and blunted associations between HAIR and hematinic indices. Once adjusted for supplement use however, reciprocal relationships between HAIR and hemoglobin/serum iron persisted. Of 568 individuals using iron tablets, most reported problems completing the course. For patients with hereditary hemorrhagic telangiectasia, persistent anemia was reported three-times more frequently if iron tablets caused diarrhea or needed to be stopped.

**Conclusions/significance:**

HAIR values, providing an indication of individuals’ iron requirements, may be a useful tool in prevention, assessment and management of iron deficiency. Iron deficiency in HHT can be explained by under-replacement of nosebleed hemorrhagic iron losses.

## Introduction

Iron deficiency is one of the most common nutritional deficiencies, globally affecting more than one billion people [1] including 12% of US females aged 12–49 years. [2] In 2002, the World Health Organisation estimated that 0.8 million (1.5%) of deaths worldwide and 35 million healthy life years lost (2.4% of global DALYs) were attributable to iron deficiency. [3] Iron has multiple essential roles, being present in oxygen binding-molecules such as hemoglobin and myoglobin, cytochromes, and many intracellular enzymes. Low serum iron concentrations provoke a hepcidin-dependent homeostatic response to co-ordinately increase intestinal iron absorption and release iron from intracellular stores, [4] but the latter are depleted in chronic iron deficiency which develops when iron demands and losses are not met by iron intake. Demands are highest during growth and pregnancy; losses are usually due to bleeding of which the most prevalent worldwide are menstruation, gastrointestinal bleeding due to hookworm, and urinary blood loss from schistosomiasis. [2] [3] Normal menstrual losses reaching 60–80 mls per month [5] [6] are why the recommended dietary iron intake for pre-menopausal females is higher than for post-menopausal females and males: recommended dietary allowances (RDA) are 18 mg/day and 8 mg/day respectively in the US. [2] Inadequate dietary iron intake is common in populations whose diets are predominantly based on starchy foods with low meat intake, [3] but is also common in developed countries: In the UK, dietary surveys indicate that up to 50% of women consume less than the national recommended iron intake for males. [7] [8] Despite this, current guidelines for management of iron deficiency anemia do not emphasise poor dietary intake as an identifiable cause of iron deficiency precluding the need for further investigations [9].

Transfusional requirements and circulatory demands that result from iron deficiency anemia are the best recognised of the diverse detrimental consequences from iron deficiency: [1] [2] [3] [10] [11] Anemic patients unable to sustain normal hemoglobin concentrations have hyperdynamic circulations, with high cardiac output accompanied by lower systemic vascular resistance. [12] Additional sequelae of iron deficiency include suboptimal immune, skeletal muscle and thyroid function; prematurity; poor maternal and perinatal outcomes in pregnancy; and impaired motor and cognitive development in children. [1] [2] [3] [10] Recently, new disease associations of iron deficiency have been recognised, including accentuation of hypoxic pulmonary hypertension, [13] and in hereditary hemorrhagic telangiectasia (HHT), association with venous thromboemboli (VTE), attributable to elevated plasma levels of coagulation factor VIII, a VTE risk factor in the general population [14].

Individuals with HHT are commonly iron deficient, generally ascribed to chronic blood loss from gastrointestinal and nasal telangiectasia. [14] [15] [16] [17] [18] More than half have visceral arteriovenous malformations (AVMs), when they need to generate supra-normal cardiac outputs to compensate for blood flow through hepatic [18] and/or pulmonary [19] AVMs. The additional burden from anemia is emphasised by the largest prospective hepatic AVM series: [18] seven of the eight cases of high output cardiac failure were precipitated by iron deficiency anemia. [18] This mirrors findings in patients from the general population with impaired cardiac reserve due to ischemic heart disease [20] [21].

The concepts that iron deficiency is insufficiently assessed and managed, [22] and not addressed by all therapies used to improve the oxygen carrying capacity in blood (such as erythropoeisis stimulating agents [23]), have been emphasised recently for the general population. [22] [23] If anemia develops, diet alone is considered inadequate as a source of iron; [22] treatment is with oral iron supplements, and if insufficient or poorly tolerated, iron infusions and/or blood transfusions. [24] [25] [26] To supplement dietary intake, the World Health Organisation recommends 60 mg/day of elemental iron to prevent iron deficiency anemia in at-risk individuals, and 120 mg per day for iron deficient individuals in 2–3 divided doses. [3] There are recognised dose-dependent side effects of iron tablets, which were best demonstrated in a 1966 study of 1,496 subjects receiving iron tablets at conventional doses (180–222 mg elemental iron/day) or placebo. [27] Calculations performed for the current study indicate that side effects were reported by 26.2% [95% CI 23.5,28.8] of iron users compared to 13.2% [10.1, 16.3] in placebo groups (p<0.0001), with tablet discontinuation due to side effects in 7.6% [6.0, 9.3] and 2.2% [0.8, 3.5] respectively (p<0.0001) [27].

Strategies to optimise iron intake focus on achieving the RDA, and treatment of existing anemia, when the iron deficit to be replaced can be formally calculated utilising iron stores and hemoglobin. [22] Specific groups at-risk of iron deficiency (individual disease cohorts and pregnant women), have iron levels and or hematinic indices monitored, but guidance from the Center for Disease Control (CDC) and elsewhere is that no routine screening for iron deficiency is recommended for men or postmenopausal women [28] [29].

Many individuals have higher iron demands than normal, and this provides the potential for a targeted, informed approach for prevention and/or treatment of iron deficiency at earlier stages, even before anemia has developed. This may also be helpful for individuals where ferritin, hemoglobin, and/or blood volume are affected by concurrent pathologies such as inflammation [30] [31] or hypoxia. [19] [32] [33] The use of pre-emptive iron supplements without proven iron deficiency is controversial, as evident from discussions on management of blood donation (which lowers iron stores [34] [35]), with concern expressed because of the potential risk of iron overload. [36] Except for menstruation and pregnancy, recommendations on additional dietary iron intake are vague, and not stratified according to those with the greatest iron losses. [1] [3] [9] [10] [11]. As emphasised by Hallberg however, [36] iron store kinetics imply it is difficult to very develop iron overload by consuming diets with high dietary iron content, unless there is a concurrent iron storage disorder. Additionally, enhanced dietary intake is not reported to cause similar side effects to iron tablets.

We hypothesised that a stratified metric which defined dietary iron requirements according to quantified hemorrhagic losses, may be helpful in assessment, prevention and treatment of iron deficiency anemia.

The HHT population potentially offered an ideal group in which to evaluate such a metric against concurrent dietary iron intakes, since nosebleeds, being both evident and usually external, can be measured to quantify hemorrhagic iron losses. [14] [15] [16] [17] [18] [37] It was therefore important to formally establish why HHT patients are iron deficient. Epistaxis (nosebleeds) is not mentioned in current guidance on evaluating iron deficient individuals, [9] although 60% of the general population experience nosebleeds, [38] [39] reflecting the superficial and easily traumatised multidirectional arterial anastomotic system in the nose. [40] Significant gastrointestinal bleeding affects relatively small proportions of individuals with HHT. [15] [16] [17] [18] We therefore also tested the hypothesis that iron deficiency may be aggravated in HHT patients due to perturbed regulation of the iron regulatory hormone hepcidin. [4] Plasma hepcidin concentrations should be lower in the setting of iron depletion, but are inappropriately elevated in several disease states including pulmonary arterial hypertension [41] which affects a small subgroup of HHT patients with mutations in *ACVRL1/*ALK1 [16] [17] [42].

The study aims, to develop a metric that stratifies recommended dietary iron intake to compensate for non-menstrual hemorrhagic losses, and evaluate whether iron deficiency can be attributed to under-replacement of hemorrhagic iron losses in HHT, were achieved: Here we present the hemorrhage-adjusted iron requirement (HAIR), that, together with hepcidin/ferritin relationships and hematinic validations, establish HHT as a model of hemorrhagic iron deficiency.

## Materials and Methods

### Ethics Statement

Named institutional review boards or ethics committees specifically approved this study: The dietary and nosebleed study was given a favorable Ethics opinion by the South West London REC3 Research Ethics Committee (11/H0803/8). All participants provided written informed consent. The survey study, named “HHT and other medical conditions” (NRES 12/EM/0073), was given a favorable Ethics opinion by the NRES Committee East Midlands- Derby 1 Research Ethics Committee on 2nd February 2012. All participants provided written or online informed consent.

### Literature Searches

At the outset of this study, it had been anticipated that an existing method to stratify iron intake according to quantified hemorrhagic losses would be available, but Pubmed searches for the terms {“iron” “intake” “hemorrhage” and either “adjust” or “stratify”} repeated most recently on 26.06.2013, identified no relevant articles other than a Cochrane review of iron intake in pregnancy. More general searches were performed using the terms “iron” and “deficiency,” initially filtered by “Practice Guidelines” or by “Systematic Review”. These identified 47 English language articles that had been published since 1992. All were reviewed for potential relevance to the current study. Three were not relevant. 25 articles referred to the management of iron deficiency in specific disease settings, characterised by non-hemorrhagic states of renal failure (n = 11); childhood (n = 5); helico pyloridus infection (n = 4); Crohn’s disease (n = 1), bariatric surgery (n = 1); thalassemia (n = 1); anemia of chronic disease (n = 1); and cancer (n = 1). An additional five articles referred to evidence for cancer screening (n = 2) or diagnostic yields from enteroscopy; colonoscopy; or gastrointestinal bleed investigations (1 each). Pubmed searches were expanded to “iron” “deficiency” “guidelines.” This retrieved 330 English language articles published since 1976, and 131 in the last 5 years. Again, most referred to management in pregnancy, renal disease, pediatric populations, cancer and guideline development/compliance. It was concluded that there were no appropriate existing indices, and the HAIR metric was devised.

Key data and opinion articles indicate that in hemodynamically tolerated iron deficiency anemia, optimal management with oral iron is generally favoured to avoid parenteral therapy. [22] [25] A Pubmed search for iron tablets side effects identified 180 articles, most referring to pregnancy or poisoning, but we were aware from our clinical practice of the difficulties non-pregnant patients experience when using iron tablets as prescribed. Published strategies to reduce gastrointestinal side effects include calculating the iron deficit to be replaced, [22] and use of different preparations, [22] [25] [27] [43] [44] [45] [46] [47] lower dosages, [22] [45] or wider dose-spacing. [22] [45] However, the best primary data on iron tablet side effects were found in the 1966 Hallberg paper, [27] for which no online Abstract or data were available. This manuscript was obtained from the British Library archives, and the published data re-analysed for presentation in a format more accessible for a modern readership. The article was also used for earlier source references.

UK service costings for investigations were obtained from national costing reports. [48] [49] In the UK, colonoscopies cost £577, an average computerised tomography scan £100–172, [48] and out-patient appointment costs averaged £147 (IQR £101, £171) [49].

### Developing the Hemorrhage Adjusted Iron Requirement (HAIR)

The hemorrhage-adjusted iron requirement (HAIR) was calculated as the sum of the normal recommended dietary iron intake, and requirements to compensate for non-menstrual blood losses. To replace normal menstrual losses, [5] [6] [46] premenopausal females are advised to consume substantially more iron than post-menopausal females or men: In the US, the Recommended Dietary Allowance (RDA) for premenopausal women is 18 mg/day, increasing to 27 mg/day when pregnant, compared to 8 mg/day for men and post-menopausal women. [2] Menstrual losses average 35–50 ml/month, but may reach 60–80 ml/month in the normal setting. [5] [6] Abnormal menstrual flow (menorrhagia) is defined at flows exceeding 80 ml/month. The additional increment of daily dietary iron recommended for non-pregnant, pre-menopausal women was therefore taken to represent that required for 80 ml of blood losses per month. In the US, this “pre-menopausal increment” equates to (18–8)mg/day = 10 mg/day, [2] though the principle can be extrapolated to any set of recommended nutrient allowances.

Blood losses are often acute, but dietary compensation takes place over many weeks. Using the paradigm of blood donation recommendations, where blood is usually donated no more than once every 3 months, and the recently validated epistaxis severity score, [50] the HAIR formula covers an equivalent 3 month period for rectification of losses. If there was a single blood loss in a 3 month period, for example following surgery or blood donation, then blood losses for months 2 and 3 would be zero:
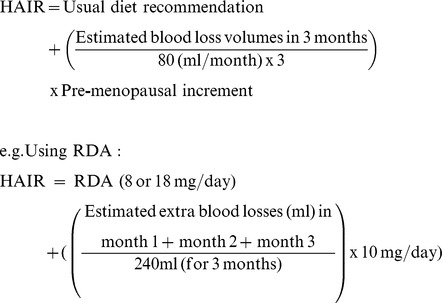



The usual dietary recommendation is determined by whether the individual is a premenstrual female with the higher RDA (or country-specific equivalent). For personalised HAIR calculations for the 50 participants in the validation survey, nose bleed volume losses per month were calculated from minutes of bleeding per month, which were converted to volumes per month according to reported intensity, and a survey of timed nose bleeds of known volume (see below).

The calculations were then repeated for published average volume losses in common clinical settings, [51] [52] [53] based on the clinical rule of thumb that one unit of blood raises the hemoglobin by 1 g/dl in the non-bleeding adult patient [51].

### HHT Diet and Nosebleed Questionnaire Study

#### Study design

A study to formally capture dietary intake in relation to hemorrhagic losses in hereditary hemorrhagic telangiectasia (HHT) was designed in late 2010, incorporating the newly published Epistaxis Severity Score. [50] Inclusion criteria were a definite diagnosis of HHT [54] and ability to give written, informed consent. Only one person per household could participate.

#### Population recruitment

Potential participants were recruited between 18^th^ April and 3^rd^ September 2011, primarily through the Imperial College London HHTIC London Clinical Service, either during attendance at clinic (N = 25), or by post, targeting the 44% of individuals in the clinic database described by Livesey et al [14] with a recorded ferritin <20 µg/L, or serum iron of less than 7 µmol/L (N = 24). One individual was also recruited by advertisement through the UK Telangiectasia Self Help website on the same page (http://www.telangiectasia.co.uk/) as the dietary iron advice sheet available since 2002. Ethical approval permitted recruitment of international HHT patients through the Kemer HHT Meeting in April 2011, but dietary intakes proved too different to food items in the EPIC FFQ to permit analyses, and the three Turkish dietary/epistaxis data sets were not included. 62 consenting British individuals provided basic demographic information and were given study questionnaires: all 50 (81%) who completed and returned their questionnaires were included in this study.

#### Quantification of nosebleed losses

Nosebleed losses were quantified using Epistaxis Severity Score (ESS) methodology. [50] Participants provide characteristics of their typical nosebleeds within the previous three months stratified by typical frequency, duration and intensity. Tick box options provided for typical frequency were less than monthly, once per month, once per week, several per week, once per day, or several per day. Options provided for typical duration (in minutes) were less than 1, 1–5, 6–15, 16–30 or more than 30. Options provided for intensity were “typically pouring or gushing” or “typically not pouring or gushing”. The ESS score incorporates three further tick box questions regarding medical attention for nose bleeds, current anemia, and transfusional requirements, and has been validated as an objective measure of nosebleed severity. [50] The current study utilised raw ESS data of typical frequency per month, typical duration, and typical intensity. To calculate blood volume lost per month, average nosebleed duration (in minutes) was multiplied by average number of bleeds per month, and by the rate appropriate to the reported intensity of bleeding. The ESS nosebleed intensity descriptions of “typically pouring or gushing” or “typically not pouring or gushing” effectively distinguish arterial and non arterial bleeding. There were no published data that formally captured the rate of nasal blood loss in these settings, although two of the dietary study participants reported nosebleeds of 270 ml in 30 minutes (9 mls/minute), and 1500 ml in approximately 2 hours (12.5 mls/minute) respectively. A larger dataset of timed nosebleeds of known volume was obtained through the Imperial 2012 HHT Survey described below. For conversions, the rate assigned for “typically pouring or gushing” nosebleeds was 7.9 mls per minute, and the rate for “typically not pouring or gushing” was 2.3 mls per minute (for derivations of these values, see below).

#### Dietary iron intake

Dietary iron intake was quantified using the European Prospective Investigation into Cancer (EPIC) food frequency questionnaire. [55] Questions are asked about the frequency of consumption of 130 food items over the previous year, methods of cooking, and use of dietary supplements. Raw data were entered into a validated EPIC software programme, the Compositional Analysis of Frequency Estimates (CAFE), to give estimated dietary iron intake. [56] This robust dietary intake assessment method has good intra-rater reliability [57].

#### Blood samples

Blood indices were measured concurrently (n = 39) or at the closest timepoints measured in the clinical service (n = 9). These measured the serum hemoglobin (Hb), hematocrit, mean corpuscular volume (MCV), mean corpuscular hemoglobin (MCH), mean corpuscular hemoglobin concentration (MCHC), red cell distribution width (RDW), serum iron, serum transferrin saturation index (T*f*SI), and serum ferritin. In addition to the above clinic assessments, 21 individuals (12 males and 9 females) consented to have research blood sampling which measured bioactive hepcidin peptide [4] by competitive radioimmunoassay [58].

### Imperial 2012 HHT Survey

To capture large numbers of nosebleed volume/time relationships in HHT, an online survey was conceived by CLS and HF during the summer of 2011. Recognising the overlapping demographic questions for same group of respondents, methodologies available through SurveyMonkey, and potential benefits of pooling questionnaires to reduce bias, CLS incorporated the nosebleed questions, and iron tablet tolerance questions relevant to the current study, into a wider survey. Following ethical approvals, the study went live on line on 6th April 2012, accessed through a specially designed Imperial College entry page at www.imperial.ac.uk/medicine/HHTsurvey2012. Potential participants were recruited through the Imperial College London HHTIC London Clinical Service databases by post; during attendance at the HHT clinics; and by advertisement by the HHT Foundation International. [37] As recently demonstrated for nosebleed precipitants [37] and antiplatelet/anticoagulant therapies, [59] subsections of the survey addressed different aspects of health and treatments for people with HHT and general population controls. Answer and question logic was applied so that individuals were only directed to questions relevant to them. Overall, of 172 questions, most respondents were directed to between 52 and 107 questions.

To obtain the data to determine the rates of blood loss in HHT nosebleeds, respondents with HHT were asked “If you have ever measured how much blood you lost in a timed bleed (“when you knew how long it lasted) please tell us here:” Reported Imperial and US cup measurements for nosebleeds of known duration were converted to mls via metricconversions.org; specific filled containers such as proprietary cups or cans via known volumes, and where hemoglobin drops over a matter of hours were available, a drop of of 1 g/dl was estimated as 750 mls of blood by back calculation from cross match requirements for surgical blood losses [51].

Additional general and HHT demographic question survey responses which were included in the current study were age, gender, and for HHT-affected respondents, whether they had required any specialised treatment for HHT, with tickboxes for specialised treatments for nosebleeds, skin telangiectasia, gastrointestinal/gut bleeding, pulmonary AVMs, cerebral AVMs, or hepatic AVMs. Those stating they had received specialised nosebleed treatments were directed to a question with tickboxes for the most common specialised treatments for HHT nosebleeds [37].

Any survey respondent who stated they had used iron tablets (general population controls or HHT) was directed to non-biased questions about tolerability, commencing with the question “If you have used iron tablets, we want to know if these were easy to take, or if they seemed to cause any problems. Please tick all that apply. When I took iron tablets………” Participants were provided with tick box options for (in order) i) “They were always fine to take and caused no problems”; ii) “They were sometimes fine to take, but sometimes seemed to cause problems”; iii) “I was able to complete the course/continue with the iron as recommended”; iv) “I had to switch to a different type of iron supplement”; v) “I had to stop taking them”; vi) “I felt sick (nauseous)”; vii) “I had pains in my stomach”; viii) “I was constipated”; ix) “I had diarrhoea”. A subsequent question asked “Have you still been anaemic despite using iron supplements?” Participants were provided with tick box options for (in order) i) “Yes”; ii) “No”; iii) “I’m not sure”, and iv ) “None of these options are applicable to me.”

For the purposes of the current study, 90 day survey data were downloaded on 12.6.2012. At the time of data download, of 913 individuals starting the survey, 756 (82.8%) had completed all of their questions. Responses for respondents who declared that they did not personally have HHT but had blood relatives with HHT were only used as general population controls if there was no declaration of HHT-related symptoms elsewhere in their survey answers: 42 self-declared controls were reclassified as being of unknown status, though none of these had used iron tablets. Overall, 569 respondents stated they had used iron tablets, and 568 (521 with HHT and 47 general population controls) provided responses to the subsequent questions on tolerability and, for HHT respondents, persistent anemia.

### Statistical Analyses

#### Power calculations

For the dietary study, pre-study power calculations using blood indices for which standard deviations were available (haemoglobin, [60] iron, [61] and transferrin saturation index [61]) indicated that 48 recruits would provide 90% power to detect clinically significant differences in these parameters between 24 participants stratified by higher and lower iron intakes, using a two group t test with a 0.05 two sided significance level. For the HHT survey, pre study power calculations were based on the most difficult endpoints to differentiate (differences in specific medical pathologies between HHT patients and controls), resulting in the aim for 1000 HHT responses, of whom only a proportion would be iron users.

#### Additional statistical details

All analyses were performed using STATA IC version 11 (Statacorp, Texas), including distributions of participant-specific variables, and two way comparisons between groups by Spearman rank correlation and Mann Whitney. Two way data plots presented individuals’ data ordered by HAIR requirements, with relevant plots superimposed. Multivariate adjusted regression coefficients and odds ratios were calculated using stepwise linear, logistic or quadratic regression: Logarithmic and inverse transformations were used to identify the most appropriate dependent variables for regression.

For the approximate quintile distributions of HAIR, stratification was made blinded to all other study parameters, and utilised naturally occurring breaks between clusters to determine the appropriate cut-offs. This resulted in four groups being larger or smaller than the true quintile size of n = 10: Quintile 1∶8.7–10.4 mg/day (n = 12); Quintile 2∶14.0–24.2 mg/day (n = 13); Quintile 3∶35.3–56 mg/day (n = 9); Quintile 4∶114–121.5 mg/day (n = 10) and Quintile 5∶195–979 mg/day (n = 6).

For the survey data, variables entered into the logistic regression model for persistent anemia if iron tablets were taken were: age; gender; indications of hemorrhagic severity (need for any nosebleed specialised treatment, that is nosebleed packs, cauterisation, laser therapy, septal dermoplasty, Youngs procedure, nasal arterial ligation, or nasal arterial embolisation; or treatments for gastrointestinal hemorrhage); other aspects of HHT status (previous treatment of skin telangiectasia, pulmonary AVMs, hepatic AVMs, or cerebral AVMs); need to stop iron tablets; need to switch iron tablets; and iron tablet generation of diarrhoea, abdominal pain, constipation or nausea. The least significant variable was removed stepwise using the p values generated by logistic regression until p = 0.10: subsequently, p values were calculated post estimation, both by likelihood ratio tests (which assume independence of observations within a cluster, an assumption that was not met with these variables), and the non parametric Wald test which does not make such assumptions.

## Results

### HAIR Calculations Reveal Substantial Dietary Iron Shortfalls in HHT Study Participants

#### Dietary iron intake in relation to normal dietary recommendations

The 50 dietary study participants comprised 28 males, and 22 females (17 post-menopausal, 5 pre-menopausal), aged 20–80 (median 55) years. Detailed intake distributions for more than 130 specified food items over the previous year are reported in Table S1. Food items included meat and fish products (17 types); bread and savory biscuits (5 types); breakfast cereals (porridge or other cereals, for which the names of the two most frequently ingested were provided); potatoes, rice, and pasta (10 types); dairy products or fats (19 types, with details about cooking methods); sweets and snacks (18 types); soups, sauces and spreads (8 types); drinks (15 types); fruits (11 types); and vegetables (26 types).

**Figure 1:**
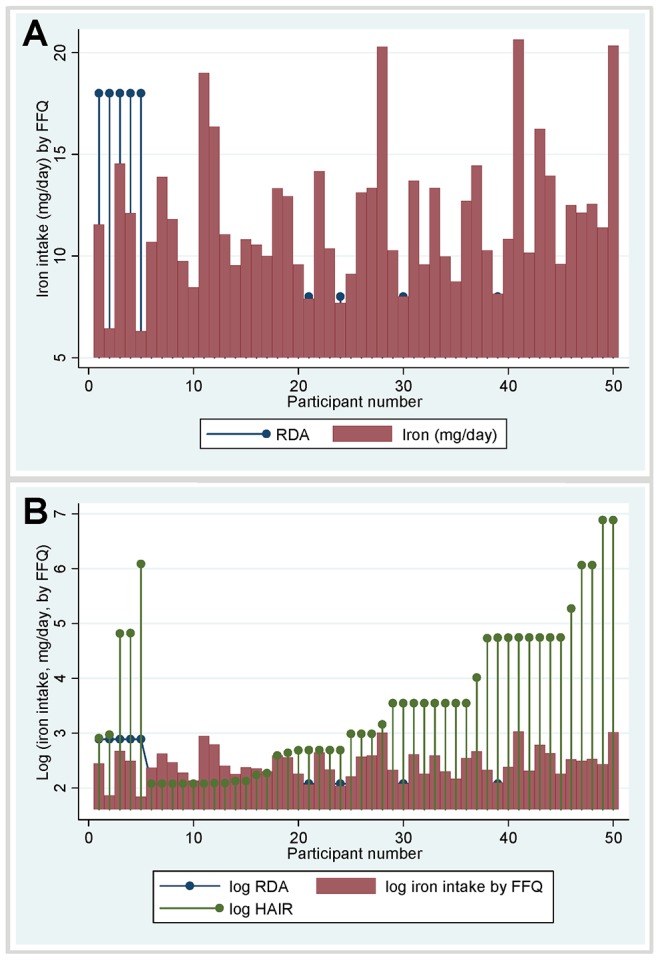
Actual and recommended dietary iron intakes for the 50 study participants. **A) Current recommendations** using US recommended dietary allowance (RDA [2]) values for iron (blue dropped line circles, at 8 or 18 mg/day): the left hand five datasets, with higher RDA values, represent the five premenopausal women, the remaining 45 datasets with lower RDA values represent males and post menopausal females. Red columns indicate each individual’s iron intake per day from their personalised food frequency questionnaire (FFQ, intake of 130 food items presented in Table S1). Note that the RDA was not met by any of the pre-menopausal females. **B) HAIR recommendations:** The same intake data as in A) are now illustrated on a natural logarithmic scale to allow presentation of each individual’s personalised HAIR value, calculated according to their personalised iron losses, and US based recommended dietary allowance (RDA) for iron, presented in Table 1. Note that a log(HAIR) of 3 corresponds to a HAIR of 20 mg/day (approximate needs of a male blood donor); a log(HAIR) of 4 to a HAIR of 55 mg/day (approximate needs over 3 months to replace a 3–4 g/dl drop in hemoglobin), and a log(HAIR) of 5 to a HAIR of 148 mg/day. Generally short (0.5–2.5 min) nosebleeds less than once per month resulted in log(HAIR) of approximately 2; several nosebleeds per week of 5 minutes or more in a log(HAIR) of ∼3; daily 10 min nosebleeds a log(HAIR) of ∼4, and several nosebleeds per day, each lasting 2.5–10 minutes, in a log(HAIR) of 5.

The diets provided a total iron intake ranging from 6.3–20.6 (median 11.3, interquartile range 9.6–13.3) mg of iron per day (Table 1). With these diets, 43 of the 50 (86%) study participants met their personal recommended intake (RDA) of 8 or 18 mg of iron per day, based on their menstrual status (Figure 1A). However, iron intake was no higher in pre-menopausal females; none of the pre-menopausal females reached their RDA of 18 mg of iron per day; and overall, only four of the study participants met the higher RDA for pre-menopausal females.

**Table 1:** Demographics and personal HAIR calculations for the 50 dietary study participants.

*Binary demographic variables*	*N*	*%*	*(95% CI)*
Gender, male	28	56	(41.8,70.3)%
Post-menopausal females	17	34	(20.4, 47.6)%
Pre-menopausal females	5	10	(1.4, 18.6)%
Achieving RDA for iron, overall	40	80	(68.5, 91.5)%
Males/Post-menopausal females (n of 45)	40	88.9	(79.3, 98.4)%
Pre-menopausal females (n of 5)	0	0%	(0)
ESS3: Nosebleed quality gushing or pouring	17	34	(20.4, 47.6)%
ESS4: Medical attention for nosebleeds	23	46	(31.7, 60.3)%
ESS5: Currently anemic	13	26	(13.4, 38.6)%
ESS6: Transfused for nosebleeds	12	24	(11.7, 36.3)%
***Continuous demographic variables***	***Median***	***Range***	***[interquartile range]***
Age (years)	55	20–80	[48.8, 67.3]
Iron intake overall (mg/day)	11	6.3–20.6	[9.6, 13.5]
ESS1: Number of nosebleeds (per month)	17.7	0–70	[3.5, 70]
ESS2: Nosebleed duration (mins)	2.5	0.5–23	[2.5, 10]
ESS Final score* (units)	4.4	0–9.1	[2.6, 5.4]
***Calculated losses and requirements***	***Median***	***Range***	***[interquartile range]***
Nosebleed losses (ml per month), median, range (IQR)	276.7	0–12,719	[21.0, 1,398]
Extra dietary iron requirements (mgs/day), median, range (IQR)	21.1	0–970	[1.6, 107]
HAIR (mg/day), median, range (IQR)	29.1	8.0–978	[13.4, 115]
Dietary iron shortfall (mg/day), median, range (IQR)	17.0	−10.3–967	[0.008, 103]

**Figure 2:**
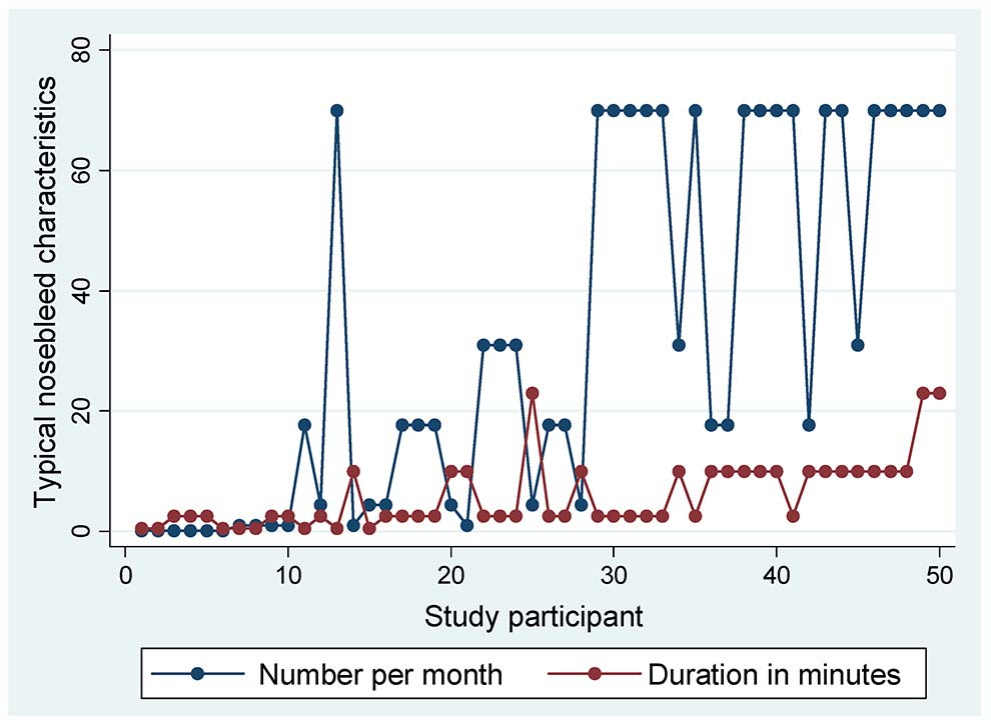
Raw data on nosebleed frequency and duration. Typical number of nosebleeds per month (blue symbols/lines, data from ESS question 1), and typical duration of nosebleeds per month (red symbols/lines, data from ESS question 2) reported by the 50 study participants, ordered by increasing value of HAIR. Nosebleeds reported as “typically gushing or pouring” were significantly longer than nosebleeds reported as “typically not gushing or pouring” (mean [standard deviation] 8.9 [6.4], versus 4.5 [4.9] minutes, Mann Whitney p = 0.0038).

General, dietary intake and nosebleed demographics and derivatives, calculated from the FFQ (Table S1) and raw data for nosebleeds (see Figure 2). RDA, recommended dietary allowance (8 mg for males and post-menopausal females, 18 mg for non pregnant pre-menopausal females); 95% CI, 95% confidence intervals; ESS, epistaxis severity score, where number refers to ESS question number. [50] *The final ESS score ranges from 0–10, where a higher score equates to greater blood losses. Dietary iron intake was no higher in pre-menopausal females (Kruskal Wallis *p* value 0.22), none of the pre-menopausal females achieved their recommended iron intake, and the difference in the proportion of pre-menopausal females and males/postmenopausal females meeting their RDA was statistically significant (p<0.001, Mann Whitney). Male gender weakly correlated with a higher dietary iron intake, but once corrected for gender, individuals with longer nosebleeds tended to have higher dietary iron intakes (data not shown).

#### Quantification of additional nosebleed hemorrhagic iron losses

In addition to usual iron demands, the HHT study participants needed to replace iron lost through HHT bleeds. They reported an average of 17.7 nosebleeds per month (interquartile range 3.5–7), with an average duration of 2.5 minutes (interquartile range 2.5–10; Table 1, Figure 2.) 17 of the 50 (34%) stated their nosebleeds were usually gushing or pouring in nature, indicative of a higher rate of blood loss.

**Figure 3:**
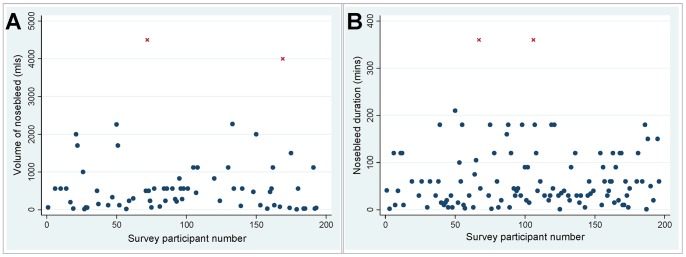
Details of the nosebleeds reported by the online survey respondents. **A)** Reported volume (mls) of individual nosebleeds, converted where appropriate from original units of measurement to mls as described in the methods. **B)** Reported duration (minutes) of individual nosebleeds. Corroborating evidence for specified major bleeds was provided by 16 individuals, and included acute hemodynamic consequences (faints, collapses, n = 5); hematocrit/hemoglobin falls (n = 4 including 3.2 g/dl hemoglobin fall in 8 hours; 8 units of hematocrit over 3 days); and unspecified acute transfusions or hospital admission (n = 8). There was no corroboratory evidence for the two indicated outliers (red crosses) whose values were excluded from calculations for the median, 20^th^ and 5^th^ percentile values used in nosebleed rate conversions**.**

To derive blood loss rates in HHT, a larger dataset was obtained through the online HHT Survey. Of the 756 survey respondents at the time of data download, 141 reported either duration (n = 112) or volume (n = 67) for specific HHT nosebleeds, and 38 (5%) reported both indices for the same nosebleed (Figure 3) The median volume lost was 473 mls (interquartile range 100, 560 mls). The survey nosebleeds were of longer duration (median 40 minutes [interquartile range 20, 90 minutes]) than the typical nosebleeds reported by the 50 dietary study participants (p<0.0001), suggesting individuals in the online survey were more likely to record and report timed volumes for their more severe nosebleeds. The median rate of blood loss (excluding the two outliers indicated in Figure 3) was 7.9 mls/minute (interquartile range 4.7, 16.7 mls).

To convert dietary study nosebleeds to volume loss per month, the median survey rate of 7.9 mls/min was used for nosebleeds described as “typically gushing,” and 20th percentile value of 2.3 ml/minute for nosebleeds described as “typically not pouring/gushing.” Using these conversion calculations, the 50 dietary study participants reported median losses of 277 mls of blood per month from their nose bleeds (interquartile range 21–1398 mls per month). Further details of these demographics are presented in Table 1.

#### Quantification of additional iron required to replace nosebleed hemorrhagic iron losses

The hemorrhage-adjusted iron requirement was calculated for each individual by summing the RDA determined by their menstrual status (8 or 18 mg/day), and additional requirements for their nosebleed losses in mls per month. Their resultant hemorrhage adjusted iron requirements (HAIR) averaged 29.1 mg of iron per day (interquartile range 13.4, 115 mg/day).

Of the 50 study participants, only 10 (20%) met their HAIR by diet alone. To illustrate graphically, individual HAIR values were plotted for each study participant. As shown in Figure 1B, this emphasised shortfalls in dietary intake for individuals who could realistically meet their HAIR through diet (generally those with typical nosebleeds occurring at most, several times per week). The graphical representation also emphasises those with extreme shortfalls due to nosebleeds once or several times per day, when iron supplements would be essential to replace hemorrhagic iron losses.

**Figure 4:**
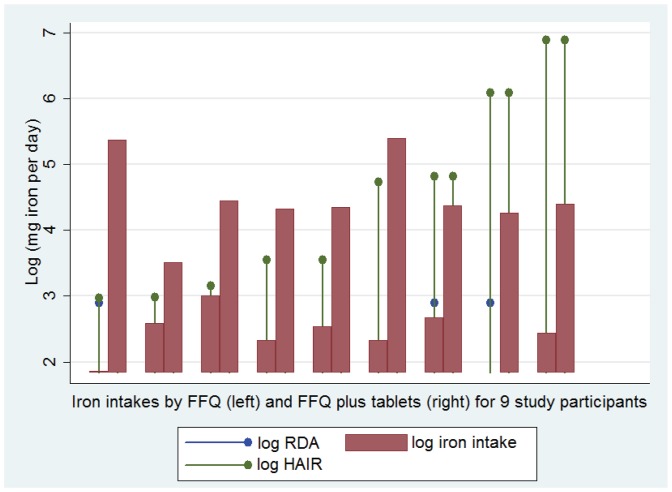
Iron intakes from diet and iron supplements. The nine individuals using ferrous sulphate, ferrous gluconate, ferrous fumarate or other iron supplements, are illustrated with pairwise comparisons of their dietary iron intake from the FFQ (left red bar) and dietary FFQ intake plus their supplement iron intake (right red bar) in addition to each individual’s log(RDA) and personalised log(HAIR), which were calculated according to their personalised iron losses and RDA for iron. As in Figure 1, note that generally short (0.5–2.5 min) nosebleeds less than once per month resulted in log(HAIR) of approximately 2; several nosebleeds per week of 5 minutes or more in a log(HAIR) of ∼3; daily 10 min nosebleeds a log(HAIR) of ∼4, and several nosebleeds per day, each lasting 2.5–10 minutes, in a log(HAIR) of 5. The two highest intakes were seen in individuals using ferrous sulphate 325 mg bd; the next six in users of once daily ferrous sulphate or ferrous fumarate.

Nine study participants were using pharmaceutical iron supplements (with elemental iron contents ranging from 20 to 65 mg) once or twice daily. The respective elemental iron content was added to dietary intakes for pairwise comparisons. Figure 4 illustrates the resultant substantial increases in daily iron intake, and that six of these nine individuals now met their HAIR.

### HAIR Validation: Prediction of Hematinic Indices in HHT

**Figure 5:**
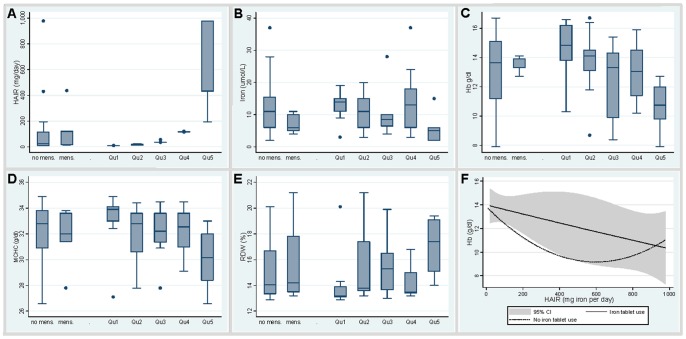
Stratification of blood hematinic and iron indices according to HAIR values and iron supplement use. **A–E:** Distributions of all participants, either by conventional recommended dietary allowance (RDA, left two box plots (mens., menses distinguishing males and post menopausal women from pre-menopausal women), or by approximate quintile (Qu) determined by HAIR (right five box plots, exact figures provided in methods). **A)** HAIR values (mg of iron per day). **B)** Serum iron (µmol/L). **C)** Hemoglobin (Hb, g/dL)). **D)** Mean corpuscular hemoglobin concentration (MCHC, g/dl). **E)** Red cell distribution width (RDW). **F)** Quadratic regression plots for the distribution of hemoglobin according to HAIR, in study participants using oral iron supplements (continuous line with 95% confidence interval indicated), and those who did not (dotted line).

In the HHT study group, there was no difference in HAIR values according to the menstrual status determining the recommended dietary allowance, or RDA (Figure 5A). There was also no relationship between RDA, age, or gender with hematinic or iron indices (data not shown).

For HAIR assessments, in order to illustrate trends, participants were stratified into approximate quintiles of iron requirements (see methods). HAIR values in the first quintile were similar to the RDA for males and post menopausal females; HAIR values in the second quintile similar to the RDA for premenopausal females. In contrast to the relationships with the standard recommended dietary allowance (RDA), stratifying participants by HAIR quintiles revealed clear trends in serum iron indices (Figure 5B). Trends were also apparent for hematinic indices such as hemoglobin (Figure 5C), mean corpuscular hemoglobin concentration (Figure 5D), and red cell distribution width (Figure 5E).

To more formally evaluate the relationships between HAIR and iron/hematinic indices, stepwise quadratic regression analyses were performed using log-transformation where appropriate to generate more normally distributed dependent variables (Figure S1). In age and gender-adjusted quadratic regression, higher HAIR was a powerful predictor of lower log-transformed serum iron (p = 0.009), lower hemoglobin (p = 0.009), higher red cell distribution width (p<0.001), and other hematinic parameters (Table 2).

**Table 2:** Quadratic regression of hematinic and iron indices with HAIR.

	*Age and gender-adjusted*			*Age, gender and iron supplement-adjusted*
*Variable*	*Coefficient (95% CI)*	*Pseudo R^2^*	*p value*	*p value*
Hemoglobin	−0.0038 (−0.0066, −0.001)	0.12	0.009	0.009
Hematocrit	−0.000062 (−0.0005, 0.000022)	0.08	0.15	0.15
MCV	0.0023 (−0.0074, 0.012)	0.002	0.64	0.73
MCH	−0.0060 (−0.012, −0.00055)	0.03	0.032	0.74
MCHC	−0.0049 (−0.0074, −0.0025)	0.1	<0.001	0.032
ln(RDW)	−0.00022 (−0.0000355, −8.15e-06)	0.09	0.002	0.0971
ln(iron)	−0.00081 (−0.0014, −0.00021)	0.09	0.009	0.009
ln (T*f*SI)	−0.00103 (−0.0016, −0.00046)	0.1	0.001	0.001
ln(ferritin)	−0.0021 (−0.0014, 0.00095)	0.01	0.72	0.72

Age and gender-adjusted HAIR regression coefficients with the designated variable were calculated using the designated variable as the dependent variable (distributional graphs are provided in Figure S1) for quadratic regression, and simultaneously examining the relationships with age, gender, and HAIR. MCV, mean corpuscular volume; MCH, mean corpuscular hemoglobin; MCHC, mean corpuscular hemoglobin concentration; ln(RDW), log-transformed red cell distribution width; ln(iron), log-transformed serum iron; ln(T*f*SI), log-transformed transferrin saturation index; ln(ferritin), log-transformed serum ferritin. 48 datasets were available except for RDW (n = 46), iron and T*f*SI (n = 45), and ferritin (n = 41). Excluding outliers in the MCV, MCH and MCHC regressions did not materially alter the results (p = 0.76 for MCV, n = 44; p = 0.035 for MCH, n = 43 and p = 0.001 for MCHC, n = 36). Age, gender and iron supplement-adjusted HAIR regression coefficients with the designated variable were calculated by quadratic regression, using the designated variable as the dependent variable, and simultaneously examining the relationships with age, gender, iron supplement use, and HAIR. Iron supplements contributed significantly to the final models of MCHC and ln(RDW), reducing the respective HAIR p values in age, gender and iron supplement adjusted regression.

There was a trend for hematinic and iron indices in participants with higher HAIR values using oral iron supplements to more closely resemble indices from participants who bled less but did not use iron supplements (Figure 5F). However, relationships between higher HAIR and hemoglobin/iron persisted after adjustment for iron supplement use (Figure 5F; Table 2). This was despite the fact that previous blood transfusions had been received by 12 participants (four of the oral iron supplement users, and eight of the study participants not using iron supplements).

### Hepcidin Levels are Appropriate for Iron Stores in HHT

**Figure 6:**
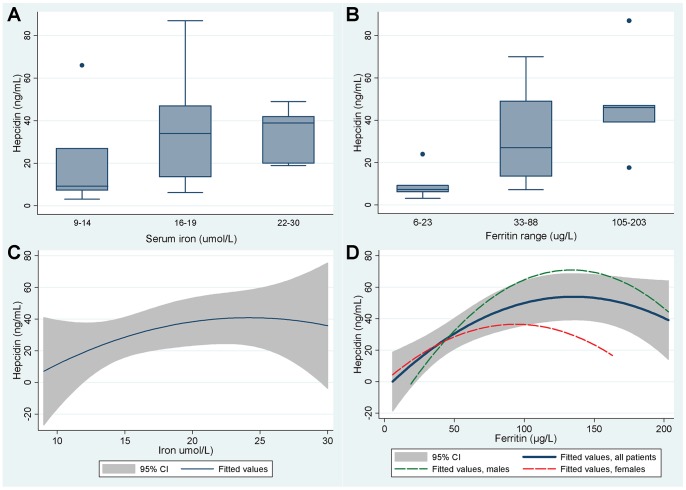
Relationships between hepcidin and iron indices. **A and B)** Hepcidin levels according to approximate tertile groupings of **A)** serum iron, and **B)** serum ferritin. Note that the reference range for hepcidin using this radioimmunoassay is 1.1–55 ng/mL. [58] Details of individual participants are provided in Table 4. **C)** and **D)** Best fit quadratic regression relationships (with shaded areas indicating the 95% confidence intervals) for hepcidin with **C)** serum iron, and **D)** ferritin.

21 of the dietary study participants consented to additional research blood sampling for hepcidin analyses. These 12 males and 9 females aged 20–69 (median 41) years displayed a wide range of iron indices and hematinics (Table 3). Normal iron metabolism handling relationships predict that hepcidin should be lower in the setting of lower iron stores, [4] and in these 21 individuals, hepcidin levels appeared to be lower in patients with lower serum iron and ferritin concentrations (Figure 6).

**Table 3:** Distribution of variables in 21 patients (12 males, 9 females) participating in iron regulatory study.

*Demographic*	*Normal range*	*Median*	*Range*	*IQR*	*P value with (ln)* *hepcidin* †
Age (years)	–	41.0	20–69	23, 58	0.39
Iron (µmol/L)	M 9–29; F 7–27	17.0	9–30	14,19	0.76
Transferrin saturation index (%)	20–45	29.0	12–46	26, 29.8	0.86
Ferritin (µg/L)	M 20–300; F 10–120	54.0	6–203	33,88	–
Hepcidin (ng/mL)	2–55	24.0	3.1–87	9.2, 46	–
Hepcidin:Ferritin ratio	‡	0.5	0.1–1.2	0.3, 0.6	n/a
Soluble transferrin receptor (sTfR),nmol/L)	8.7–28.1	21.5	13.2–44.1	19.1, 24.5	0.59
sTfR/log Ferritin (Thomas ratio)		1.0	0.5–4.8	0.8, 1.5	0.32
Hemoglobin(g/dL)	M 12.5–17; F 11.4–15	14.3	12.2–16.7	13.9, 15.9	0.51
Mean corpuscular volume, MCV (fL)	83–101	87.8	77.8–97.8	84.6, 90.3	0.69
Creatinine (µmol/L)	M 60–125; F 60–110	74.0	59.-99	66, 81	0.61
eGFR (mL/min/1.73 msq)	>59	87.0	9–95	76, 82.7	0.93
Albumin(g/L)	33–47	41.0	33–49	40, 43	0.24

M, males; F, female**;** eGFR, estimated glomerular filtration rate; n/a, not appropriate.

†indicates p value on separate addition to final model for hepcidin, once adjusted for ferritin and ferritin^2^.

‡For the control group reported in [63] the geometric mean was 0.68 (95% confidence intervals 0.41, 0.96). In contrast, Piperno et al reported upper 95% confidence intervals of the means for hemachromatosis heterozygotes and homozygotes as <0.4 and <0.2 respectively [63].

To evaluate formally, stepwise multiple regression were performed, using log transformed (ln)hepcidin as the dependent variable because this displayed a more normal distribution than hepcidin. As shown in Table 4A, 60% of the variance in ln(hepcidin) was explained by a model incorporating only ferritin (p<0.0001) and its higher order variable, ferritin^2^ (p = 0.001). This proportion of 60% is almost identical to the proportion of hepcidin variance explained by ferritin levels in the control population reported by Busbridge et al. [58] The bulk of the remaining variance (32.4% of the total variance in hepcidin levels) was explained by the hepcidin:ferritin ratio (Table 4B). This ratio is reduced in patients with liver disease [62] (hepcidin is synthesised in the liver), [4] and modified in iron storage disorders such as hemachromatosis. [63] In the HHT cohort, there was a wide range of hepcidin:ferritin ratios (Table 2). Importantly, the geometric mean and 95% confidence intervals (0.49 [95% confidence intervals 0.35, 0.62]) corresponded to the hepcidin:ferritin ratio values reported for the control group studied by Piperno et al. [63] Since the relationships between ferritin and hepcidin in the HHT patients were very similar to those reported for general population controls, [58] [63] we concluded there was no evidence to suggest a generalised abnormality of iron handling in the HHT cohort.

**Table 4:** Multiple regression of log transformed (ln) hepcidin.

*Variable*	*Regression coefficient (95% CI)*	*Standard error*	*p value*
**A) ** ***Ferritin restricted***			
Ferritin	0.04 (0.023, 0.058)	0.0083	<0.0001
Ferritin^2^	−0.00015 (−0.00028, −0.000066)	0.000039	0.001
**B) ** ***Complete model***			
Ferritin	0.036 (0.028, 0.044)	0.0037	<0.001
Ferritin^2^	−0.00011 (−0.00015, −0.000076)	0.000017	<0.001
Hepcidin:ferritin	1.88 (1.43, 2.34)	0.22	<0.001

**A)** Model using ferritin alone. 95% CI, 95% confidence intervals. Overall model parameters: sum of squares 17.2; 20 degrees of freedom; mean square 0.86; variance ratio (F) 11.0; adjusted r^2^ = 60.0; overall p value for model = 0.0001. **B)** The final model for ln(hepcidin), generated by stepwise multiple regression in data from 21 HHT patients. Overall model parameters: sum of squares 17.2, 20 degrees of freedom, mean square 0.86, variance ratio (F) 81.5, adjusted r^2^ = 92.4, overall p value for model <0.0001. No other captured variable contributed to the model.

### A Role for HAIR Even if Iron Supplements are Prescribed

Eight study participants who had previously been transfused were not using iron supplements, 46/50 (92%) study participants could have met their HAIR using twice daily oral iron supplements (Figure 1),and HHT patients frequently reported poor tolerance of iron tablets in clinic. These data prompted a formal assessment of iron tablet tolerability which was performed as part of the online survey used to obtain timed nosebleed data. Of the 568 respondents who stated that they had used iron tablets, barely half reported that iron tablets were always or sometimes fine to take (280/521 HHT patients (53.7%), 28/47 healthy controls (59.6%), p = 0.44). Very high proportions reported constipation (218/568, 38.3%); nausea (131/568, 23.1%) or diarrhea (60/568, 10.6%), with no difference between the healthy controls and HHT patients. Once adjusted for indices of HHT hemorrhage, persistent anemia was reported three times as frequently by HHT patients reporting diarrhoea, and almost four times more frequently if iron tablets needed to be stopped (Table 5). We concluded that there was a role for guidance of dietary iron intake, even if iron tablets were prescribed.

**Table 5:** Multivariate logistic regression of the risk of persistent anemia, if using iron tablets.

*Variable*	*Odds ratio (95% CI)*	*Standard error*	*z test*	*LR test p value*	*Wald test p value*
Had to stop iron tablets	3.70 (1.68, 8.18)	1.50	3.25	0.0005	0.0012
Iron tablets cause diarrhoea	3.09 (1.45, 6.62)	1.20	2.91	0.0020	0.0036
Had to switch iron tablets	2.24 (1.25, 4.02)	0.67	2.70	0.0059	0.0070
Nasal septal dermoplasty performed	2.35 (1.30, 4.27)	0.71	2.82	0.0038	0.0048
Treatment for gastrointestinal hemorrhage	3.06 (1.61, 5.81)	1.00	3.42	0.0003	0.0006
Treatment for skin telangiectasia	1.70 (1.06, 2.72)	0.41	2.21	0.0266	0.0273

The final model presenting all variables making a significant contribution to the risk of persistent anemia, once adjusted for the presence of other variables, in HHT online survey respondents using iron tablets. The model details 424 observations providing a pseudo r^2^ of 0.14, and overall model *p* value of <0.0001. P values were calculated post estimation, both by likelihood ratio (LR) tests which assume independence of observations within a cluster (an assumption that was not met with these data), and the non parametric Wald test which does not make such assumptions. There was no clear relationship between persistent anemia and iron tablet-induced nausea, constipation, or abdominal pain (likelihood ratio test *p* values 0.14, 0.11, and 0.09 respectively). There was also no relationship with age, gender, other otorhinolaryngologic treatments [37] either combined or individually, or other reported HHT treatments for pulmonary, cerebral or hepatic arteriovenous malformations (data not shown).

### Application of HAIR to Common Clinical Settings in the General Population

Having validated HAIR as a predictor of hematinic indices in the HHT population, and demonstrated that iron handling appeared to be normal in this specific hemorrhagic condition, HAIR values were calculated for blood loss volumes relevant to common clinical settings in the general population, such as post partum bleeding, [52] surgery, [51] fractures, [53] and blood donation. Table 6 illustrates the HAIR-based recommendations for iron intake for the general population, based on two different national sets of recommended dietary intakes: In the US, premenopausal women are advised to consume 18 mg/day, compared to 8 mg/day for men and post-menopausal women (RDA, as used in calculations presented in the current study). [2] In the UK, the respective iron Reference Nutrient Intake (RNI) values are 14.8 mg/day for premenopausal women, and 8.7 mg/day for men and post-menopausal women [64].

**Table 6:** HAIR values for typical clinical settings.

		*US values based on RDA, mg/day*		*UK values based on RNI, mg/day*	
*Setting*	*Approximate volume loss* *(ml)*	*Males or post menopausal females*	*Pre menopausal* *non pregnant* *females*	*Males or post menopausal females*	*Pre menopausal females*
***No additional losses***	0	8	18	8.7	14.8
***Blood donation every 3 months***	470	27.6	37.5	20.6	26.7
Hemorrhagic hemoglobin fall* of					
1 g/dl, e.g normal peri-partum loss [52]	470	27.6	37.6	20.6	26.7
2 g/dl e.g. TURP; colectomy, minor postpartumhemorrhage [52]	940	47.2	57.2	32.6	38.7
3 g/dl e.g. moderate post partum hemorrhage [52]	1410	66.8	76.8	44.5	50.6
4 g/dl e.g. nephrectomy [51]	1880	86.3	96.3	56.5	62.6
***Femoral head fracture *** [***53***]	611	33.5	43.5	24.2	30.3

HAIR values were calculated by the formula given in the Methods, correcting over 3 months. *HAIR values can be extended to incorporate existing deficits manifest by hemoglobin falls, although other guidance exists in this setting; [22] these figures exclude any hemorrhagic losses and hemoglobin falls that are corrected by transfusions, since the transfused red cell iron content remains available for body stores. TURP, transurethral resection of the prostate. The reasons for the differences between columns are that the recommended dietary iron intake for premenopausal females is substantially higher than for post-menopausal females and for men, and because normal dietary intake recommendations vary between countries: In the UK, the respective iron Reference Nutrient Intake (RNI) values are 14.8 mg/day and 8.7 mg/day [64]. In the US, premenopausal women are advised to consume 18 mg/day, increasing to 27 mg/day when pregnant, compared to 8 mg/day for men and post-menopausal women (recommended dietary allowance, RDA, as used in calculations presented in the current study). [2] UK HAIR values are therefore lower than US-based HAIR values because of the lower recommendations for premenopausal women, and because the UK increment of 6.1 mg/day applies instead of the US increment of 10 mg/day for premenopausal women. The principles and calculation can be extended to any other set of dietary allowances.

## Discussion

In this study, an innovative approach and new metric revealed extreme shortfalls in dietary iron intake that were not apparent if dietary iron intake was only assessed using usual iron dietary requirements. In the study population with HHT, iron-deficient hematinics were predicted by the hemorrhage-adjusted iron requirement (HAIR). Since serum hepcidin concentrations were appropriate for ferritin levels, a generalised derangement in iron handling seems highly unlikely, suggesting that iron deficiency in HHT can be attributable purely to under-replacement of hemorrhagic losses. Because the HAIR validations were performed in a rare disease population, this may suggest that HAIR is relevant only to a relatively narrow audience of clinicians caring for HHT patients, but this is not the case. The concepts are generic, and we simply exploited the quantifiable hemorrhagic losses of nosebleeds to provide data that has been undeveloped for many decades in internal medicine [5] [6] [46].

The strengths of the overall study included selection of a single patient cohort with evident and quantifiable chronic hemorrhagic iron losses; use of the currently accepted differential for dietary iron intake to compensate for menstrual blood losses; participant awareness of the need for iron-rich diets (evidenced by the higher proportion meeting their recommended dietary intake than other UK studies [7] [8]); and the study participants’ marked hemorrhagic iron losses which allowed a clear differential between their ability to meet usual recommended dietary intakes, and HAIR. The HHT survey benefited from targeting a group with high proportions requiring long term iron therapy, and highly motivated to participate in research studies as evidenced by the rapid response rate.

The survey of self-reported tolerance or iron tablets and anemia is the weakest component of the current study, particularly as it did not include a placebo group for medications where Kerr and Davidson elegantly demonstrated suggestibility of side effects. [65] The data are included because they provide a further large series that suggests high proportions of patients have to limit or modify their iron treatments, and that poor toleration of iron tablets is associated with persistent anemia. Since actual blood loss from nosebleeds may be greater than stated due to hemorrhaged blood that is swallowed, the nosebleed-based estimation may be an underestimation for some patients. A minor weakness of the study was that the rate of each individual bleeding event was not available, and instead calculated from patient reports of timed nosebleed volumes, applied according to reported intensity which effectively distinguishes arterial and non arterial bleeding. Importantly, using even the 5th percentile value (0.3 mls per minute) in conversion of all nosebleed volumes, still resulted in only 22 individuals meeting the resultant partially-adjusted iron requirements. The proportion of dietary iron absorbed differs in unpredictable ways according to factors such as genetic variability in iron handling genes, iron status/hepcidin levels, [4] and co-existent dietary intake of iron absorption modifiers such as phytates, oxylates and polyphenols. [36] By basing the HAIR calculation on the currently accepted differential for iron intake to compensate for menstrual blood losses, we were able go some way towards bypassing the uncertainties of individual iron absorption rates, but this would be useful to address in future studies.

We suggest that the HAIR metric will be particularly helpful for predictive recommendations, for patients in whom ferritin and hemoglobin are affected by pathologies other than iron deficiency, and as an accessible tool to use in clinic. It is very difficult to consume diets containing 20 mg of iron per day without careful dietary planning: in the current study the greatest dietary iron intake achieved was 20.6 mg/day. Figure 1 suggests patients with nosebleeds occurring less than daily (HAIR values <20 mg/day; log (HAIR) <3) may be reasonably expected to achieve their extra iron intake through diet alone, but daily nosebleeds are likely to require formal iron supplements, whether or not there are any additional hemorrhagic losses. This is important for clinicians as epistaxis is not considered an important clinical question when evaluating iron deficient individuals. The rich nasal plexi of vessels required for the physiological warming and humidification of inspired air run superficially within the nasal mucosa, and are easily traumatised by dry, cold inflow, finger damage, and constituents of nasal sprays. Nosebleeds are common [38] and are more frequent and prolonged for individuals with hypertension, coagulopathies or using anti-platelet [39] and anti-coagulant therapies. There is a tendency to assume that chronic anemia-precipitating hemorrhage must be from occult gastrointestinal bleeding. But the superficial blood supply of the nose is a rich multidirectional arterial anastomotic system, [40] thus representing a significant potential site of bleeding, not captured by current medical histories.

Within the HHT population, clearly there will be some individuals where HAIR will offer lesser corrective opportunities because of chance co-inheritance of concurrent iron handling defects. There have also been theoretical HHT-specific concerns regarding aberrant hepcidin regulation, but it is intriguing to note that hepcidin perturbations would operate in different directions: Inappropriately high hepcidin concentrations are found in pulmonary arterial hypertension [41] which is usually caused by mutations in *BMPR2*, but also affects a small subgroup of HHT patients with mutations in *ACVRL1/*ALK1 [42]. Furthermore, the proteins mutated in HHT patients can associate with bone morphogenetic protein receptors (including BMPR2), that bind BMP6, a master regulator of hepcidin. [4] However hepatic arteriovenous malformations are also more common in the ACVRL1/ALK1 HHT patient subgroup. [16] [17] [18] While liver synthetic function is generally preserved, in late stage hepatic AVM disease, abnormalities in synthetic function and frank cirrhosis may occur, [16] [17] [18] and would be predicted to reduce the hepcidin:ferritin ratio as seen in other forms of liver disease/cirrhosis [62].

For the general population, we suggest HAIR could also play important roles in the prevention and management of iron deficiency as it does not require blood test results for calculation: The CDC does not recommend routine iron screening blood tests for men or postmenopausal women [28] [29], but symptomatic presentations and incidental discovery carry attendant health care burdens - for example, iron deficient patients are more likely to require unplanned transfusions following elective surgery. [24] Prospective application of the HAIR concept may therefore be helpful for both clinicians and patients to reduce later morbidity. HAIR may also have a retrospective role, in the assessment of iron deficiency of unknown cause: In current guidance, poor dietary intake is not always emphasised as an identifiable cause of iron deficiency precluding the need for further gastrointestinal investigations to identify sources of occult blood loss. [31] This means that patients may be referred for expensive investigations when a simple assessment of iron intake and subsequent advice may resolve both the cause and the deficiency. To address whether adoption of HAIR will reduce iron deficiency rates and/or invasive investigation costs, we suggest three randomised trials. In the first, large post surgical cohorts could be prospectively randomised to receive standard management, standard dietetic advice, or dietetic advice incorporating HAIR values relevant to their quantified surgical losses. The second could use a similar approach to determine if HAIR calculations are useful for prevention or treatment of iron deficiency due to menorrhagia, which remains an important cause of iron deficiency in young women. This would require estimation of excessive blood losses beyond normal menses, but given there is little rigorous guidance for iron replacement estimates in this clinical setting, the HAIR formula may be helpful. Additionally, comparison of gastroenterological referral rates and outcomes could be made in iron deficient patients randomised to either standard referral practices, or an initial HAIR-based dietary assessment.

In summary, hereditary hemorrhagic telangiectasia is confirmed as a model of hemorrhagic iron deficiency, where nosebleed/iron intake comparisons may be used to predict the severity of iron deficient hematinic indices. There is a role for dietary guidance, even if iron tablets are prescribed. We anticipate that wider application of the HAIR metric, allowing clinicians to prospectively adjust recommended iron intake to compensate for hemorrhagic losses, may improve understanding, and reduce the wider clinical and financial burdens of this very common nutritional deficiency.

## Supporting Information

Table S1 **Portions of food items per day.** Breakdown of dietary intake in preceding year for 130 food items, as reported by the 50 dietary study participants using the European Prospective Investigation into Cancer (EPIC) food frequency questionnaire (FFQ). (main article reference [55]) Questions are asked about the frequency of consumption of 130 food items over the previous year, methods of cooking, and use of dietary supplements, and are presented in food groups: meat and fish products (17 types); bread and savory biscuits (5 types); breakfast cereals (porridge or other cereals); potatoes, rice, and pasta (10 types); dairy products or fats (19 types, and details about cooking methods); sweets and snacks (18 types); soups, sauces and spreads (8 types); drinks (15 types); fruits (11 types); and vegetables (26 types). N: Number of participants (of 50) reporting item; sd, standard deviation; min, minimum; max, maximum.
(DOCX)

Figure S1 **Normal quantile plots of dependent variables used in regression analyses.** In contrast to distributions for (A) hemoglobin, and (B) hematocrit, distributions of other indices were skewed. Normalisation was achieved by logarithmic transformation of (C) iron, (D) transferrin saturation index (T*f*SI), (E) ferritin, and (F) hepcidin. Distributions of mean corpuscular volume (MCV), mean corpuscular hemoglobin (MCH), mean corpuscular hemoglobin concentration (MCHC), and red cell distribution width (RDW) remained skewed after logarithmic and/or inverse transformation: The variables with distributions most approximating to normality (G) MCV, (H) MCHC (and MCH, distribution comparable, data not shown), and (I) lnRDW were used for regression analyses, and regression results confirmed by re-testing after exclusion of outliers (data not shown).
(TIF)
