# Supplementary material for: Hemorrhage-Adjusted Iron Requirements, Hematinics and Hepcidin Define Hereditary Hemorrhagic Telangiectasia as a Model of Hemorrhagic Iron Deficiency
Source: PLoS One. 2013 Oct 16;8(10):e76516. doi: 10.1371/journal.pone.0076516 (PMC3797784; doi:10.1371/journal.pone.0076516)
Supplement: Table S1 — Portions of food items per day. Breakdown of dietary intake in preceding year for 130 food items, as reported by the 50 dietary study participants using the European Prospective Investigation into Cancer (EPIC) food frequency questionnaire (FFQ). (main article reference [55]) Questions are asked about the frequency of consumption of 130 food items over the previous year, methods of cooking, and use of dietary supplements, and are presented in food groups: meat and fish products (17 types); bread and savory biscuits (5 types); breakfast cereals (porridge or other cereals); potatoes, rice, and pasta (10 types); dairy products or fats (19 types, and details about cooking methods); sweets and snacks (18 types); soups, sauces and spreads (8 types); drinks (15 types); fruits (11 types); and vegetables (26 types). N: Number of participants (of 50) reporting item; sd, standard deviation; min, minimum; max, maximum. (DOCX) [file pone.0076516.s001.docx]

**Haemorrhage-adjusted iron requirements (HAIR) and hepcidin/ferritin relationships define hereditary haemorrhagic telangiectasia as a model of haemorrhagic iron deficiency**

**Helen Finnamore, James Le Couteur, Mary Hickson, Mark Busbridge, Kevin Whelan and Claire L. Shovlin**

**Supplementary Table: Portions of food items per day**

Breakdown of dietary intake in preceding year for 130 food items, as reported by the 50 dietary study participants using the European Prospective Investigation into Cancer (EPIC) food frequency questionnaire (FFQ).[main article reference 55] Questions are asked about the frequency of consumption of 130 food items over the previous year, methods of cooking, and use of dietary supplements, and are in food groups: meat and fish products (17 types); bread and savory biscuits (5 types); breakfast cereals (porridge or other cereals); potatoes, rice, and pasta (10 types); dairy products or fats (19 types, and details about cooking methods); sweets and snacks (18 types); soups, sauces and spreads (8 types); drinks (15 types); fruits (11 types); and vegetables (26 types). N: Number of participants (of 50) reporting item; sd, standard deviation; min, minimum; max, maximum.

| **Variable** | **N** | **mean** | **sd** | **min** | **max** |  |  | |
| --- | --- | --- | --- | --- | --- | --- | --- | --- |
| -------------+------------------------------------------------------------------------------------------------------- | | | | | |  |  | |
| *MEAT AND FISH PRODUCTS (medium serving, portions per day)* | | | | | | | |  |
| Beef | 49 | 0.207 | 0.171 | 0.000 | 0.770 |  |  | |
| Beefburgers | 49 | 0.040 | 0.114 | 0.000 | 0.770 |  |  | |
| Pork | 48 | 0.086 | 0.101 | 0.000 | 0.420 |  |  | |
| Lamb | 49 | 0.074 | 0.101 | 0.000 | 0.420 |  |  | |
| Chicken | 49 | 0.271 | 0.193 | 0.000 | 0.770 |  |  | |
| Bacon | 50 | 0.099 | 0.111 | 0.000 | 0.420 |  |  | |
| Ham | 50 | 0.218 | 0.245 | 0.000 | 0.770 |  |  | |
| Corned beef/spam | 50 | 0.052 | 0.119 | 0.000 | 0.420 |  |  | |
| Sausages | 50 | 0.088 | 0.101 | 0.000 | 0.420 |  |  | |
| Savoury pies | 50 | 0.067 | 0.089 | 0.000 | 0.420 |  |  | |
| Liver/liver pate | 50 | 0.017 | 0.033 | 0.000 | 0.140 |  |  | |
| Fried fish | 50 | 0.060 | 0.076 | 0.000 | 0.420 |  |  | |
| Fish fingers | 50 | 0.036 | 0.047 | 0.000 | 0.140 |  |  | |
| Other white fish | 50 | 0.106 | 0.121 | 0.000 | 0.420 |  |  | |
| Oily fish | 50 | 0.104 | 0.119 | 0.000 | 0.420 |  |  | |
| Shell fish | 50 | 0.050 | 0.074 | 0.000 | 0.420 |  |  | |
| Fish roe/taramasalata | 50 | 0.004 | 0.017 | 0.000 | 0.070 |  |  | |
| ---------------------------------------------------------------- | | | | | |  |  | |
| *BREAD AND SAVORY BISCUITS (one slice or biscuit, portions per day)* | | | | | |  |  | |
| White bread/rolls | 50 | 0.906 | 1.151 | 0.000 | 4.500 |  |  | |
| Brown bread/rolls | 50 | 0.400 | 0.754 | 0.000 | 4.500 |  |  | |
| Wholemeal bread/rolls | 50 | 0.638 | 1.006 | 0.000 | 4.500 |  |  | |
| Cream crackers | 50 | 0.129 | 0.186 | 0.000 | 0.770 |  |  | |
| Crisp bread | 50 | 0.091 | 0.189 | 0.000 | 0.770 |  |  | |
| ---------------------------------------------------------------- | | | | | |  |  | |
| *CEREALS, (one bowl, portions per day)* | | | | | |  |  | |
| Porridge | 49 | 0.185 | 0.312 | 0.000 | 1.000 |  |  | |
| Breakfast cereals other | 50 | 0.510 | 0.404 | 0.000 | 1.000 |  |  | |
| -------------+-------------------------------------------------- | | | | | |  |  | |
| *potatoes, rice and pasta (medium serving, portions per day)* | | | | | | | | |
| Boiled, mashed, jacket | 49 | 0.444 | 0.311 | 0.000 | 1.000 |  |  | |
| Chips | 50 | 0.158 | 0.172 | 0.000 | 0.770 |  |  | |
| Roast potatoes | 50 | 0.191 | 0.357 | 0.000 | 2.500 |  |  | |
| Potato salad | 50 | 0.036 | 0.087 | 0.000 | 0.420 |  |  | |
| White rice | 50 | 0.150 | 0.183 | 0.000 | 0.770 |  |  | |
| Brown rice | 50 | 0.042 | 0.093 | 0.000 | 0.420 |  |  | |
| White/green pasta | 50 | 0.130 | 0.130 | 0.000 | 0.420 |  |  | |
| Wholemeal pasta | 49 | 0.041 | 0.074 | 0.000 | 0.420 |  |  | |
| Lasagne/moussaka | 49 | 0.050 | 0.047 | 0.000 | 0.140 |  |  | |
| Pizza | 50 | 0.049 | 0.053 | 0.000 | 0.140 |  |  | |
| ---------------------------------------------------------------- | | | | | |  |  | |

| **Variable** | **N** | **mean** | **sd** | **min** | **max** |  |  |
| --- | --- | --- | --- | --- | --- | --- | --- |
| -------------+------------------------------------------------------------------------------------------------------- | | | | | | |  |
| *DAIRY PRODUCTS AND FATS, portions per day* | | | | | | |  |
| Single/sour cream (tbsp) | 49 | 0.049 | 0.105 | 0.000 | 0.420 |  |  |
| Double cream (tbsp) | 50 | 0.034 | 0.089 | 0.000 | 0.420 |  |  |
| Low fat yogurt (125g) | 50 | 0.240 | 0.255 | 0.000 | 1.000 |  |  |
| Full fat yogurt (125g) | 50 | 0.094 | 0.248 | 0.000 | 1.000 |  |  |
| Dairy dessert (125g) | 49 | 0.063 | 0.165 | 0.000 | 1.000 |  |  |
| Cheese (medium serve) | 50 | 0.356 | 0.275 | 0.000 | 1.000 |  |  |
| Cottage cheese (medium) | 50 | 0.104 | 0.235 | 0.000 | 1.000 |  |  |
| Eggs, cooked (one) | 50 | 0.210 | 0.179 | 0.000 | 0.770 |  |  |
| Quiche (medium) | 50 | 0.049 | 0.089 | 0.000 | 0.420 |  |  |
| Low fat dressing (tbsp) | 50 | 0.084 | 0.183 | 0.000 | 1.000 |  |  |
| Salad cream/mayo (tbsp) | 50 | 0.088 | 0.134 | 0.000 | 0.420 |  |  |
| French dressing (tbsp) | 50 | 0.056 | 0.114 | 0.000 | 0.420 |  |  |
| Other dressing (tbsp) | 50 | 0.043 | 0.103 | 0.000 | 0.420 |  |  |
| -------------+-------------------------------------------------- | | | | | |  |  |
| *THE FOLLOWING ON BREAD OR VEGTABLES, portions per day* | | | | | | | |
| Butter (tsp) | 50 | 0.529 | 0.813 | 0.000 | 2.5 |  |  |
| Block margarine (tsp) | 50 | 0.072 | 0.368 | 0.000 | 2.500 |  |  |
| Polyunsaturated (tsp) | 50 | 0.426 | 0.823 | 0.000 | 2.500 |  |  |
| Soft margarine (tsp) | 50 | 0.549 | 0.815 | 0.000 | 2.500 |  |  |
| Low fat spread (tsp) | 50 | 0.174 | 0.604 | 0.000 | 2.500 |  |  |
| Very low fat spread (tsp) | 50 | 0.093 | 0.636 | 0.000 | 4.500 |  |  |
| ---------------------------------------------------------------- | | | | | |  |  |
| *SWEETS AND SNACKS (medium serving, portions per day)* | | | | | | |  |
| Sweet biscuits, chocolate | 50 | 0.780 | 1.162 | 0.000 | 4.500 |  |  |
| Sweet biscuits, plain | 50 | 0.347 | 0.441 | 0.000 | 2.500 |  |  |
| Cakes, homemade | 49 | 0.157 | 0.250 | 0.000 | 1.000 |  |  |
| Cakes, ready made | 50 | 0.101 | 0.187 | 0.000 | 1.000 |  |  |
| Buns, pastries, homemade | 49 | 0.109 | 0.236 | 0.000 | 1.000 |  |  |
| buns, pastries, ready made | 50 | 0.134 | 0.232 | 0.000 | 1.000 |  |  |
| Fruit pie/tart, homemade | 50 | 0.060 | 0.136 | 0.000 | 0.770 |  |  |
| Fruit pie/tart, ready made | 50 | 0.039 | 0.115 | 0.000 | 0.770 |  |  |
| Sponges, homemade | 50 | 0.015 | 0.035 | 0.000 | 0.140 |  |  |
| Sponges, ready made | 50 | 0.027 | 0.086 | 0.000 | 0.420 |  |  |
| Milk pudding, homemade | 50 | 0.082 | 0.351 | 0.000 | 2.500 |  |  |
| Ice cream, choc ice | 50 | 0.182 | 0.644 | 0.000 | 4.500 |  |  |
| Chocolate, single/squares | 48 | 0.461 | 0.694 | 0.000 | 2.500 |  |  |
| Chocolate bars eg mars | 50 | 0.226 | 0.414 | 0.000 | 2.500 |  |  |
| Sweets, toffees | 50 | 0.135 | 0.215 | 0.000 | 1.000 |  |  |
| Sugar added to tea | 50 | 1.296 | 1.954 | 0.000 | 6.000 |  |  |
| Crisps, packet snacks | 50 | 0.449 | 0.627 | 0.000 | 2.500 |  |  |
| Peanuts, other nuts | 50 | 0.148 | 0.387 | 0.000 | 2.500 |  |  |
|  | | | | | | | |

| **Variable** | **N** | **mean** | **sd** | **min** | **max** |  |  |
| --- | --- | --- | --- | --- | --- | --- | --- |
| -------------+-------------------------------------------------- | | | | | | |  |
| *SOUPS, SAUCES AND SPREADS, portions per day* | | | | | | | |
| Vegetable soup (bowl) | 50 | 0.125 | 0.149 | 0.000 | 0.420 |  |  |
| Meat soups (bowl) | 50 | 0.057 | 0.104 | 0.000 | 0.420 |  |  |
| Sauses eg white (tbsp) | 50 | 0.256 | 0.282 | 0.000 | 1.000 |  |  |
| Tomato ketchup (tbsp) | 50 | 0.231 | 0.405 | 0.000 | 2.500 |  |  |
| Pickles chutney (tbsp) | 50 | 0.155 | 0.230 | 0.000 | 1.000 |  |  |
| Marmite, Bovril (tsp) | 50 | 0.114 | 0.370 | 0.000 | 2.500 |  |  |
| Jam, marmalade (tsp) | 50 | 0.493 | 0.742 | 0.000 | 4.500 |  |  |
| Peanut butter (tsp) | 50 | 0.062 | 0.178 | 0.000 | 1.000 |  |  |
| -------------+-------------------------------------------------- | | | | | | |  |
| *DRINKS, portions per day* | | | | | | |  |
| Tea (cup) | 50 | 3.094 | 2.116 | 0.000 | 6.000 |  |  |
| Coffee (cup) | 50 | 1.136 | 1.356 | 0.000 | 4.500 |  |  |
| Coffee decaf (cup) | 50 | 0.118 | 0.292 | 0.000 | 1.000 |  |  |
| Coffee whitener (tsp) | 49 | 0.049 | 0.207 | 0.000 | 1.000 |  |  |
| Cocoa/hot chocolate (cup) | 49 | 0.043 | 0.090 | 0.000 | 0.420 |  |  |
| Horlicks/ovaltine (cup) | 50 | 0.084 | 0.381 | 0.000 | 2.500 |  |  |
| Wine (glass) | 50 | 0.359 | 0.798 | 0.000 | 4.500 |  |  |
| Beer/cider (half pint) | 49 | 0.319 | 0.555 | 0.000 | 2.500 |  |  |
| Port/sherry (glass) | 50 | 0.015 | 0.064 | 0.000 | 0.420 |  |  |
| Spirits eg brandy (glass) | 50 | 0.256 | 0.922 | 0.000 | 6.000 |  |  |
| Low calorie drinks (glass) | 50 | 0.164 | 0.416 | 0.000 | 2.500 |  |  |
| Fizzy soft drink (glass) | 49 | 0.157 | 0.399 | 0.000 | 2.500 |  |  |
| Pure fruit juice (glass) | 49 | 0.392 | 0.554 | 0.000 | 2.500 |  |  |
| Apple juice (glass) | 50 | 0.206 | 0.456 | 0.000 | 2.500 |  |  |
| Fruit squash (glass) | 50 | 0.357 | 0.696 | 0.000 | 2.500 |  |  |
| -------------+-------------------------------------------------- | | | | | |  |  |
| *FRUITS (for seasonal fruits starred, estimate average use when in season, portions per day)* | | | | | | | |
| Apples (1 fruit) | 50 | 0.332 | 0.453 | 0.000 | 2.5 |  |  |
| Pears (1 fruit) | 50 | 0.153 | 0.214 | 0.000 | 1 |  |  |
| Oranges/Satsuma (1 fruit) | 49 | 0.298 | 0.445 | 0.000 | 2.500 |  |  |
| Grapefruit (half fruit) | 50 | 0.028 | 0.072 | 0.000 | 0.420 |  |  |
| Bananas (1 fruit) | 50 | 0.369 | 0.457 | 0.000 | 2.5 |  |  |
| Grapes (medium serve) | 50 | 0.227 | 0.274 | 0.000 | 1.000 |  |  |
| Melon (1 slice) | 50 | 0.085 | 0.153 | 0.000 | 0.77 |  |  |
| Peaches/plums (1 fruit) | 50 | 0.184 | 0.381 | 0.000 | 2.500 |  |  |
| Strawberries (1 fruit) | 50 | 0.196 | 0.196 | 0.000 | 0.770 |  |  |
| Tinned fruit (medium) | 50 | 0.035 | 0.068 | 0.000 | 0.420 |  |  |
| Dried fruit (medium) | 50 | 0.094 | 0.208 | 0.000 | 1.000 |  |  |
| ---------------------------------------------------------------- | | | | | |  |  |

| **Variable** | **N** | **mean** | **sd** | **min** | **max** |  |  |
| --- | --- | --- | --- | --- | --- | --- | --- |
| ---------------------------------------------------------------- | | | | | | |  |
| *VEGETABLES fresh, frozen or tinned (medium serving, portions per day)* | | | | | |  |  |
| Carrots | 50 | 0.314 | 0.190 | 0.000 | 1.000 |  |  |
| Spinach | 50 | 0.111 | 0.233 | 0.000 | 1.000 |  |  |
| Broccoli, spinach | 50 | 0.228 | 0.184 | 0.000 | 0.770 |  |  |
| Brussels sprouts | 50 | 0.077 | 0.125 | 0.000 | 0.420 |  |  |
| Cabbage | 50 | 0.106 | 0.119 | 0.000 | 0.420 |  |  |
| Peas | 50 | 0.292 | 0.363 | 0.000 | 2.500 |  |  |
| Green/broad/runner beans | 50 | 0.182 | 0.185 | 0.000 | 0.770 |  |  |
| Marrow/courgettes | 50 | 0.078 | 0.127 | 0.000 | 0.420 |  |  |
| Cauliflower | 50 | 0.109 | 0.118 | 0.000 | 0.420 |  |  |
| Parsnips/turnips | 50 | 0.106 | 0.127 | 0.000 | 0.420 |  |  |
| Leeks | 50 | 0.071 | 0.125 | 0.000 | 0.770 |  |  |
| Onions | 50 | 0.275 | 0.248 | 0.000 | 1.000 |  |  |
| Garlic | 50 | 0.231 | 0.257 | 0.000 | 1.000 |  |  |
| Mushrooms | 50 | 0.208 | 0.213 | 0.000 | 1.000 |  |  |
| Sweet peppers | 50 | 0.174 | 0.223 | 0.000 | 1.000 |  |  |
| Beansprouts | 50 | 0.041 | 0.072 | 0.000 | 0.420 |  |  |
| Green salad, cucumber | 50 | 0.348 | 0.302 | 0.000 | 1.000 |  |  |
| Watercress | 49 | 0.095 | 0.197 | 0.000 | 1.000 |  |  |
| Tomatoes | 49 | 0.436 | 0.348 | 0.000 | 1.000 |  |  |
| Sweetcorn | 50 | 0.155 | 0.175 | 0.000 | 0.770 |  |  |
| Beetroot | 50 | 0.154 | 0.218 | 0.000 | 1.000 |  |  |
| Coleslaw | 50 | 0.114 | 0.204 | 0.000 | 1.000 |  |  |
| Avocado | 50 | 0.050 | 0.089 | 0.000 | 0.420 |  |  |
| Baked beans | 49 | 0.182 | 0.209 | 0.000 | 1.000 |  |  |
| Dried lentils | 50 | 0.048 | 0.105 | 0.000 | 0.420 |  |  |
| Tofu/soya meat | 50 | 0.056 | 0.178 | 0.000 | 0.770 |  |  |
| -------------+-------------------------------------------------- | | | | | |  |  |
| *SUMMARY,*  *Portions per day of :* |  |  |  |  |  |  |  |
| Meat and meat products | 49 | 1.475 | 1.458 | 0.000 | 4.500 |  |  |
| Fish and fish products | 49 | 0.353 | 0.889 | 0.000 | 4.500 |  |  |
| Vegetables  (not potatoes) | 47 | 2.392 | 2.592 | 0.070 | 9.140 |  |  |
| Fruit and products  (not juice) | 47 | 3.008 | 3.207 | 0.000 | 11.500 |  |  |
| Breakfast cereals  (not oats) | 50 | 0.51 | 0.404 | 0.000 | 1.00 |  |  |
| ---------------------------------------------------------------- | | | | | |  |  |
