# Supplementary figures and images for: Hemorrhage-Adjusted Iron Requirements, Hematinics and Hepcidin Define Hereditary Hemorrhagic Telangiectasia as a Model of Hemorrhagic Iron Deficiency
Source: PLoS One. 2013 Oct 16;8(10):e76516. doi: 10.1371/journal.pone.0076516 (PMC3797784; doi:10.1371/journal.pone.0076516)

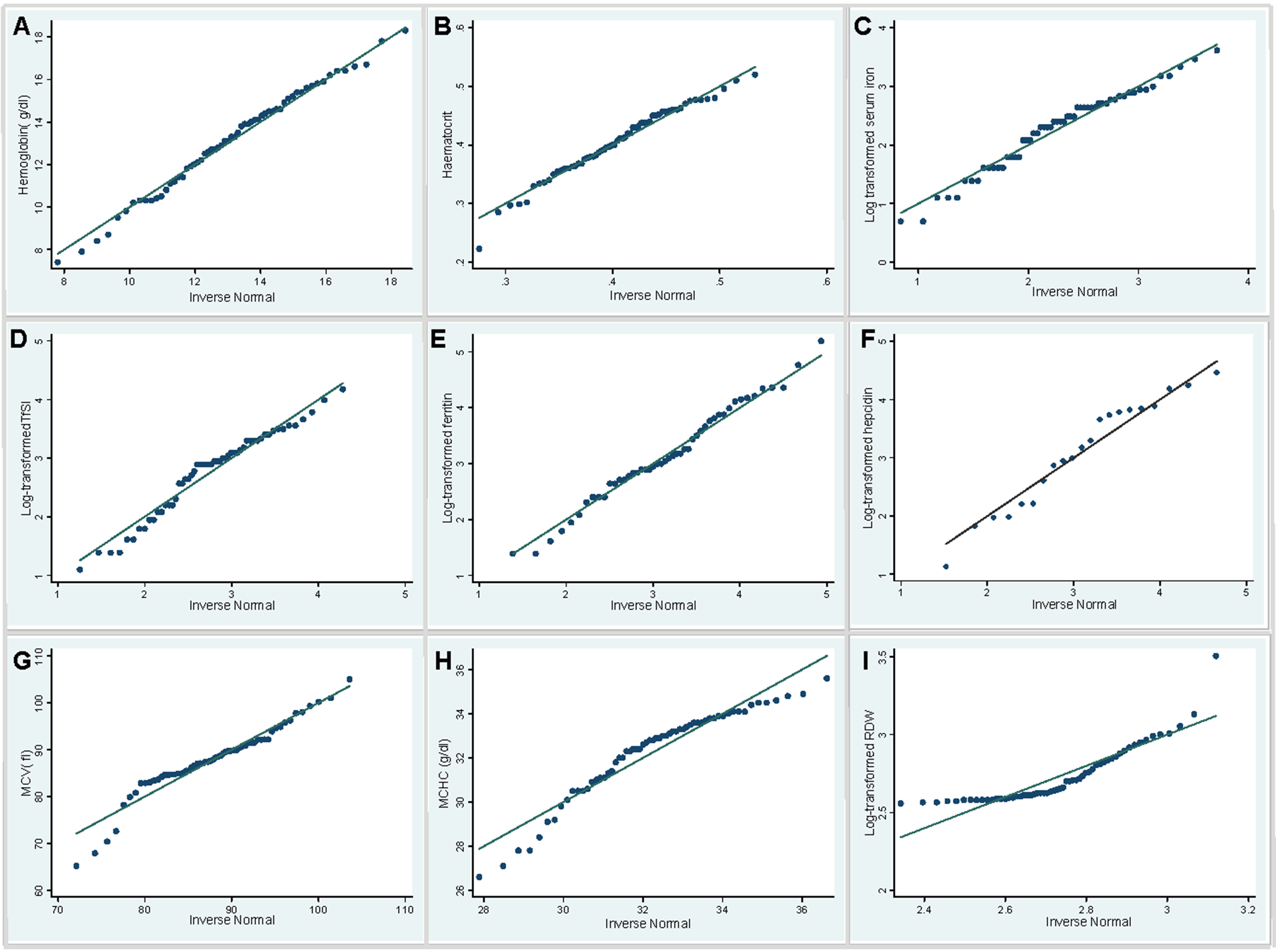

Supplement: Figure S1 — Normal quantile plots of dependent variables used in regression analyses. In contrast to distributions for (A) hemoglobin, and (B) hematocrit, distributions of other indices were skewed. Normalisation was achieved by logarithmic transformation of (C) iron, (D) transferrin saturation index (TfSI), (E) ferritin, and (F) hepcidin. Distributions of mean corpuscular volume (MCV), mean corpuscular hemoglobin (MCH), mean corpuscular hemoglobin concentration (MCHC), and red cell distribution width (RDW) remained skewed after logarithmic and/or inverse transformation: The variables with distributions most approximating to normality (G) MCV, (H) MCHC (and MCH, distribution comparable, data not shown), and (I) lnRDW were used for regression analyses, and regression results confirmed by re-testing after exclusion of outliers (data not shown). (TIF) [file pone.0076516.s002.tif]
